# Electronic epidemiological query on admission: early detection of high risk patients for pulmonary tuberculosis

**DOI:** 10.1186/2047-2994-4-S1-P61

**Published:** 2015-06-16

**Authors:** C Palos, A Bispo, P Rodrigues, V Pinto, J Mapril

**Affiliations:** 1 Infection Control and Antibiotics Committee, Hospital Beatriz Ângelo, Loures, Portugal; 2 Infection Control Committee, Hospital da Luz, Lisboa, Portugal

## Introduction

Pulmonary tuberculosis (PT) patients are a threat to healthcare workers, students, other patients and visitors. Hospitals should implement strategies in order to detect such patients early on admission, mainly on the Emergency Room (ER) and adopt adequate isolation procedures. This is especially important where prevalence or resistance is high.

## Objectives

Implementation of a simple tool allowing early detection of high risk patients for PT and adequate isolation procedures.

## Methods

The Infection Control and Antibiotics Committee (ICAC) implemented an Electronic Epidemiological Query on Admission (EEQA) using the electronic medical registry (EMR) - SOARIAN(R), Siemens Medical Solutions, Cerner. EEQA is an eight question tool filled for every patient at inhospital admission. It automatically generates adequate micro orders, isolation procedures and e-mails to ICAC, and ultimately allows detection of colonization/infection by Epidemiologically Important Microrganisms (MRSA, Carbapenem-resistant *Acinetobacter baumanii*, VRE), or infection by *Clostridium difficile* or pulmonary *Mycobacterium tuberculosis*. Regarding PT, a positive answer to the question “PT suspected or confirmed” (based on clinical judgement) generates prescriptions of respirator use and negative pressure room placement.

## Results

On year 2014, the question regarding high risk for PT was positive in 50 cases (1,42% of 3.523 positive EEQA, 35% of 10.106 ward admissions). Adequate isolation procedures were implemented on 48 of 50 positive PT EEQA (96% of positive PT risk EEQA). PT was confirmed on 13 patients (26% of suspected PT). Positive PT risk was assumed on 12 of 13 confirmed cases (92%).

## Conclusion

EEQA is a tool that detects the vast majority of PT patients, allowing early and adequate isolation procedures (respirator use, adequate ER and ward placement), starting on the ER, even before micro confirmation. This protects healthcare workers, students, other patients and visitors from exposure to these patients, thus minimizing the risk for nosocomial PT.

## Disclosure of interest

None declared.

